# Evaluation of Graphene Oxide Induced Cellular Toxicity and Transcriptome Analysis in Human Embryonic Kidney Cells

**DOI:** 10.3390/nano9070969

**Published:** 2019-07-02

**Authors:** Sangiliyandi Gurunathan, Muhammad Arsalan Iqbal, Muhammad Qasim, Chan Hyeok Park, Hyunjin Yoo, Jeong Ho Hwang, Sang Jun Uhm, Hyuk Song, Chankyu Park, Jeong Tae Do, Youngsok Choi, Jin-Hoi Kim, Kwonho Hong

**Affiliations:** Department of Stem Cell and Regenerative Biotechnology and Humanized Pig Center (SRC), Konkuk Institute of Technology, Konkuk University, Seoul 05029, Korea

**Keywords:** graphene oxide, human embryonic kidney cells, cellular assays, transcriptomic analysis, oxidative stress, apoptosis

## Abstract

Graphene, a two-dimensional carbon sheet with single-atom thickness, shows immense promise in several nanoscientific and nanotechnological applications, including in sensors, catalysis, and biomedicine. Although several studies have shown the cytotoxicity of graphene oxide in different cell types, there are no comprehensive studies on human embryonic kidney (HEK293) cells that include transcriptomic analysis and an in vitro investigation into the mechanisms of cytotoxicity following exposure to graphene oxide. Therefore, we exposed HEK293 cells to different concentrations of graphene oxide for 24 h and performed several cellular assays. Cell viability and proliferation assays revealed a significant dose-dependent cytotoxic effect on HEK293 cells. Cytotoxicity assays showed increased lactate dehydrogenase (LDH) leakage and reactive oxygen species (ROS) generation, and decreased levels of reduced glutathione (GSH) and increased level of oxidized glutathione indicative of oxidative stress. This detailed mechanistic approach showed that graphene oxide exposure elicits significant decreases in mitochondrial membrane potential and ATP synthesis, as well as in DNA damage and caspase 3 activity. Furthermore, our RNA-Seq analysis revealed that HEK293 cells exposed to graphene oxide significantly altered the expression of genes involved in multiple apoptosis-related biological pathways. Moreover, graphene oxide exposure perturbed the expression of key transcription factors, promoting these apoptosis-related pathways by regulating their downstream genes. Our analysis provides mechanistic insights into how exposure to graphene oxide induces changes in cellular responses and massive cell death in HEK293 cells. To our knowledge, this is the first study describing a combination of cellular responses and transcriptome in HEK293 cells exposed to graphene oxide nanoparticles, providing a foundation for understanding the molecular mechanisms of graphene oxide-induced cytotoxicity and for the development of new therapeutic strategies.

## 1. Introduction

Graphene oxide is a two-dimensional monolayer composed primarily of carbon atoms. It is the oxidized form of graphene and contains various oxygen-containing functionalities, such as epoxide, carbonyl, carboxyl, and hydroxyl groups. Graphene oxide has a large surface area and is cheaper and easier to manufacture than graphene [1,2]. Due to its large surface area and unique and exceptional physical, chemical, and biological properties (e.g., electronic, optical, and mechanical), graphene oxide is widely used in biomedical engineering and biotechnological applications, including antibacterial and antiplatelet applications, drug delivery, tissue engineering, diagnostics, and photothermal and photodynamic therapies [3,4,5]. The functionality of graphene oxide as either a toxic or biocompatible material depends on its composition, size, surface, shape, functional groups, charge, coating, and dissolving media [6]. Moreover, graphene oxide is frequently used in biological systems as a drug delivery agent, as well as in cellular imaging probes and biosensors because of its hydrophilic nature [7,8].

Due to the extensive range of applications for graphene oxide, cytotoxicity studies are essential for the safe and sustainable development of graphene oxide-based nanotechnologies [9]. *In vitro* assays are effective strategies as a first approach for determining the cytotoxicity of nanomaterials. Several studies have been conducted to estimate the level of toxicity in different cell types, including pheochromocytoma-derived PC12 [10], HeLa, MCF-7, SKBR3, NIH3T3, epithelial lung carcinoma, primary mouse embryonic fibroblast, human breast cancer, ovarian cancer, and HepG2 cells, and graphene oxide toxicity was found to be both dose- and time-dependent [11,12,13,14]. Graphene oxide induces cell toxicity through plasma membrane damage, generation of reactive oxygen species (ROS), and DNA damage. Using three sizes of commercially available graphene oxide and six different cell lines, Gies and Zou (2018) reported that the overall toxicity of graphene oxide varied greatly between cell lines, with suspended cells showing greater responses than adherent cells [15]. Oxidative stress has been proposed as one of the major mechanisms of nanomaterial-induced toxicity due to increased generation of reactive chemical species that play important roles in cell signaling and homeostasis [16].

Graphene oxide biocompatibility with several cell lines is dependent on the size of the particles. Graphene oxide was found to elicit toxicity only at high concentrations in human fibroblast cells (HDF); moreover, Gurunathan et al. [17] reported that graphene oxide could induce dose-dependent toxicity in mouse embryonic fibroblasts. The biocompatibility of graphene oxide can be enhanced by functionalization using surface coatings like bovine serum albumin, polyethylene glycol, dextran (DEX), and poly(amidoamine) (PAMAM) dendrimers [18]. For example, graphene oxide functionalized using a recombinant enhanced green fluorescent protein (EGFP) showed excellent biocompatibility with human kidney cells compared to graphene oxide alone [19]. Studies from several authors have claimed that graphene oxide biocompatibility also depends on the presence of reducing agents and particle size; particles with sizes ranging from 100–200 nm can be used as effective drug carriers, while particles smaller than 100 nm can induce toxicity [20]. Recently, Sun et al. [21] found that graphene oxide regulates *COX2* via epigenetic mechanisms in HEK293T cells.

Cell survival and death are two major toxicity endpoints that can potentially be affected by any nanoparticle treatment. Carbon nanoparticles, in particular, evoke severe toxicity by inducing apoptosis and mitochondrial dysfunction. Due to the extensive use of graphene oxide, it is necessary to minimize its cytotoxicity and determine the associated regulatory molecular mechanisms. Recent findings suggest that the graphene oxide treatment can impair the general cellular priming state, including eliciting disorders of the plasma membrane and cytoskeleton construction [22]. Graphene oxide has emerged as an anticancer agent and chemosensitizer; however, the detailed molecular basis underlying this graphene oxide-induced state is still unknown. To understand the molecular mechanisms involved in graphene oxide-induced toxicity, next-generation sequencing technologies would be aid in our understanding of the mechanisms involved in graphene oxide-induced toxicity. High-throughput methods like genome tiling arrays were previously used to study global transcription [23,24]. More recently, RNA sequencing analysis (RNA-Seq) has been used to map transcribed regions globally and analyze RNA isoforms quantitatively at an unprecedented level of sensitivity and accuracy [25,26].

In this study, we selected HEK293 cells as a model system to investigate the effects of graphene oxide. These cells are widely used in cell biology and biotechnology to study signal transduction and protein interactions. Non- or low tumorigenic HEK293 cells are frequently used to test the oncogenic properties of cancer-associated genes. Furthermore, these cells most closely resemble adrenal cells, which possess numerous neuronal properties. Given the broad use of HEK293 cells for biomedical research, we used HEK293 cells to investigate the comprehensive cellular effects and molecular mechanisms associated with graphene oxide toxicity.

## 2. Materials and Methods

### 2.1. Materials

HEK293 cell lines were purchased from the American Type Culture Collection (ATCC, Manassas, VA, USA). Graphite powder, NaOH, KMnO_4_, NaNO_3_ anhydrous ethanol, H_2_SO_4_, 36% HCl, and 30% H_2_O_2_ aqueous solution were purchased from Sigma-Aldrich (St Louis, MO, USA). The penicillin-streptomycin solution, trypsin-ethylenediaminetetraacetic acid solution, Dulbecco’s modified Eagle’s medium (DMEM), and 1% antibiotic-antimycotic solution were obtained from Gibco (Life Technologies, Carlsbad, CA, USA). Fetal bovine serum (FBS) and the in vitro toxicology assay kit were purchased from Sigma-Aldrich. The kits for measuring MDA and antioxidants levels were also purchased from Sigma-Aldrich, as were all other chemicals unless otherwise stated. 

### 2.2. Synthesis of Graphene Oxide

Graphene oxide was synthesized as described previously, with suitable modifications [16,27]. For a typical synthesis, 2 g of natural graphite (Gt) powder was added to 350 mL of H_2_SO_4_ at 0 °C; then, 8 g of KMnO_4_ and 1 g of NaNO_3_ were added gradually while stirring. The mixture was transferred to a 40 °C water bath and stirred for 60 min. Deionized water (250 mL) was slowly added, and the temperature was increased to 98 °C. The mixture was maintained at 98 °C for 30 min and the reaction was then terminated by adding 500 mL of deionized water and 40 mL of a 30% H_2_O_2_ solution. The color of the mixture changed to a brilliant yellow, indicating oxidation of pristine graphite to graphite oxide. The mixture was filtered and washed with dilute HCl to remove metal ions. The product was washed repeatedly with distilled water until a pH of 7.0 was achieved and then further sonicated for 15 min. To prepare smaller sized graphene oxide particles, the prepared, brownish mixture of graphene oxide was centrifuged and washed several times with deionized water. The unexfoliated graphene oxide was removed by centrifugation at 12,000 rpm for 5 min, and the as-prepared graphene oxide was dried at 40 °C. To obtain a uniform distribution of smaller sized graphene oxide sheets, a low-speed centrifugation was performed at 5000 rpm for 15 min to remove thick multilayer flakes until all the visible particles had been removed. The supernatant was then further subjected to ultrasonication for 1 h. The resulting graphene oxide samples were used for subsequent analyses.

### 2.3. Cell Culture and Treatment

HEK293 cell lines were maintained in Dulbecco’s modified Eagle’s medium (DMEM) supplemented with 10% FBS, 100 U/mL penicillin, and 100 μg/mL streptomycin in a humidified incubator in 5% CO_2_ at 37 °C. Cells were routinely grown in 100-mm plastic tissue culture dishes (Nunc, Roskilde, Denmark) and harvested at the logarithmic phase of growth using a trypsin-EDTA solution. Experiments were performed in 96-, 24-, and 12-well plates and 100-mm cell culture dishes, as indicated. Cells were treated with various concentrations of graphene oxide or the required dose of graphene oxide. Silver nanoparticles (25 μg/mL) were used as a positive control and media without cells used as a negative control. In order to verify the performance of graphene oxide, all the cellular assays experiments, we used media without cells as a negative control (NC) and silver nanoparticles (AgNPs) as a positive control. 

### 2.4. Cell Viability and Cell Proliferation Assays 

Cell viability and cell proliferation were measured using a Cell Counting Kit-8 (CCK-8) and a BrdU assay kit. Briefly, HEK293 cells were plated in 96-well flat-bottom culture plates containing various concentrations of graphene oxide. After 24 h of culture at 37 °C and 5% CO_2_ in a humidified incubator, 10 μL of a CCK-8 solution was added to each well and the plate was incubated for an additional 2 h at 37 °C. The absorbance was measured at 450 nm using a microplate reader (Multiskan FC; Thermo Fisher Scientific Inc., Waltham, MA, USA). Cell proliferation was determined according to the manufacturer’s instructions (Roche).

### 2.5. Measurement of Membrane Integrity and Dead-Cell Protease Activity 

HEK293 cell membrane integrity was evaluated using an LDH Cytotoxicity Detection Kit according to the manufacturer’s instructions. Briefly, cells were exposed to various concentrations of graphene oxide for 24 h, following which dead-cell protease activity was assessed using a previously described method [28]. The cytotoxicity assay was used to evaluate the cytotoxic effects of graphene oxide on HEK293 cells. Cytotoxicity was determined based on the reaction of intracellular proteases with a luminogenic peptide substrate (alanyl-alanyl-phenylalanyl-aminoluciferin). Luminescence was measured using a Luminescence Counter (Perkin Elmer, Waltham, MA, USA).

### 2.6. Determination of Intracellular ROS Levels 

HEK293 cells were treated with various concentrations of graphene oxide for 24 h. ROS levels were measured according to a previous method based on the intracellular peroxide-dependent oxidation of 2′,7′-dichlorodihydrofluorescein diacetate (DCFH-DA, Molecular Probes, Waltham, Massachusetts, USA) to form the fluorescent compound, 2′,7′-dichlorofluorescein (DCF) [29].

### 2.7. Measurement of MDA Content and Antioxidant Enzyme Activities

Antioxidant enzyme activities and MDA content were measured using a previously described method [16]. HEK293 cells were treated with different concentrations of graphene oxide for 24 h, and the MDA content was then determined using the thiobarbituric acid method. Measurement of antioxidant enzyme activities was performed according to the manufacturer’s instructions. For the MDA and antioxidant enzyme assays, cell cultures were washed twice with PBS, scraped from the plates into ice-cold PBS (0.1 M, containing 0.05 mM EDTA), and homogenized. The homogenate was centrifuged at 10,000 rpm for 15 min at 4 °C and the resulting supernatant was stored at 70 °C until analysis. Protein concentration was determined using the Bradford method, with bovine serum albumin (BSA) as the reference standard.

### 2.8. JC-1 Assay 

HEK293 cells were treated with various concentrations of graphene oxide for 24 h. Changes in MMP were determined using the cationic fluorescent dye, JC-1 (Molecular Probes, Eugene, OR, USA). Fluorescence of JC-1 aggregates and JC-1 monomers was measured at an excitation wavelength of 488 nm and emission wavelengths of 583 and 525 nm, respectively, using a Gemini EM fluorescence microplate reader (Molecular Device, Sunnyvale, CA, USA).

### 2.9. Measurement of ATP Levels

ATP levels were measured in HEK293 cells according to the manufacturer’s instructions (Catalog Number MAK135; Sigma-Aldrich). The cells were exposed to various concentrations of graphene oxide for 24 h and the ATP level was then measured.

### 2.10. Measurement of 8-oxo-dG 

8-oxo-dG and its analogs were used as biomarkers of oxidative DNA damage and oxidative stress. To evaluate graphene oxide-induced oxidative stress in HEK293 cells, an 8-oxo-dG ELISA assay was utilized as previously described [30] and following the manufacturer’s instructions (Trevigen, Gaithersburg, MD, USA).

### 2.11. Measurement of Caspase 3 Activity

Caspase 3 activity was measured according to previously described methods [31]. Cells were treated with various concentrations of graphene oxide for 24 h, and then the activity of caspase 3 was measured using a kit (Sigma-Aldrich) according to the manufacturer’s instructions. This calorimetric assay was based on the hydrolysis of the caspase 3 substrate by caspase 3, resulting in the release of the p-nitroaniline (pNA) moiety. The concentration of the pNA released from the substrate was calculated from the absorbance values at 405 nm. The assay was performed using non-induced cells as a control.

### 2.12. RNA-Seq and Bioinformatics

Total RNA was isolated from both untreated and AdNP-treated HEK293 cells using an RNeasy kit (Qiagen). Before the RNA extraction, the cells were thoroughly washed with ice-cold PBS to remove dead cells. The RNA concentration and quality were measured on a bioanalyzer using Bioanalyzer RNA chips (Agilent technologies, Santa Clara, CA USA). Total RNAs with ˃7 RNA integrity number (RIN) were used for library preparation. An RNA-Seq library was prepared using an Illumina TruSeq Stranded total RNA sample preparation kit following the manufacturer’s instructions. Approximately 1 µg of total RNA was reverse transcribed. The synthesized cDNA was then subjected to fragmentation, adaptor ligation, PCR amplification, and gel size selection. Sequencing was performed using a NextSeq500 sequencer (Illumina, San Diego, CA USA). After sequencing, the reads were assessed for quality and mapped to the National Center for Biotechnology Information (NCBI) Human Genome Assembly (hg19) using FastQC and the STAR pipeline, respectively. Using the Cufflink tool, differentially expressed genes (DEGs) were determined using a cutoff value (FPKM) >3 and a fold change (FC) >3. The DEGs were then subjected to gene ontology (GO) term analysis using DAVID, and the results were plotted using GOplot (MLL Münchner Leukämielabor GmbH, Munich, Germany). Scatter plot and GOplot images were generated in R (v3.3.2).

### 2.13. Enrichment, Pathway, and Transcription Factor Analyses of the DEGs

GSEA (v3.0, BROAD Institute, Cambridge, MA, USA) was used to determine the biological enrichment in the DEGs of the treatment group. For this, gene expression data in GCT format, molecular signature database (MsigDB) in GMT format, and phenotype labels in CLS file format were used. KEGG pathway enrichment analysis for the DEGs from the graphene oxide-treated HEK293 cells was performed using the ClueGO plug-in (v2.5.1, INSERM, Paris, France) for Cytoscape (v3.6.1, Institute of Systems Biology, Seattle, WA, USA). ClueGO generates functionally clustered KEGG annotation networks with the DEGs. The *p*-value was calculated by right-sided hypergeometric tests and Benjamin–Hochberg adjustment was used for multiple test correction. KEGG pathways with a *p*-value < 0.05 were considered significant. Coexpression and physical interactions (protein–protein) of the DEGs were determined using GeneMANIA plug-in (v3.5.0, University of Toronto, Ontario, Canada) for Cytoscape. A query-dependent weighting method for the selected genes was used in the analysis.

### 2.14. Statistical Methods

Independent experiments were repeated at least three times with three replicates for each concentration. Data were analyzed by a Student’s *t*-test to determine the one sample to control and significant differences denoted by an asterisk. The results are presented as mean ± standard deviation of three experiments. *p < 0.05 was considered significant, **p < 0.01 was considered highly significant, and ***p < 0.001 was considered very highly significant.

## 3. Results and Discussion

### 3.1. Synthesis and Characterization of Graphene Oxide

**Figure 1:**
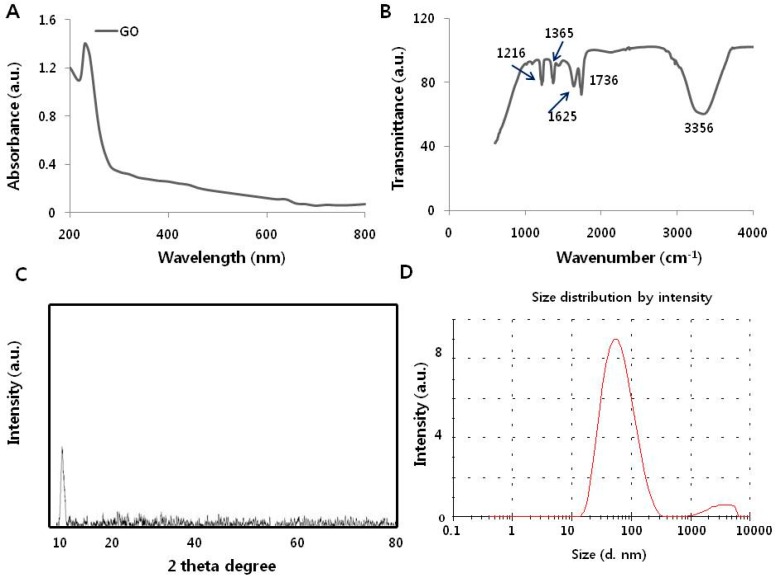

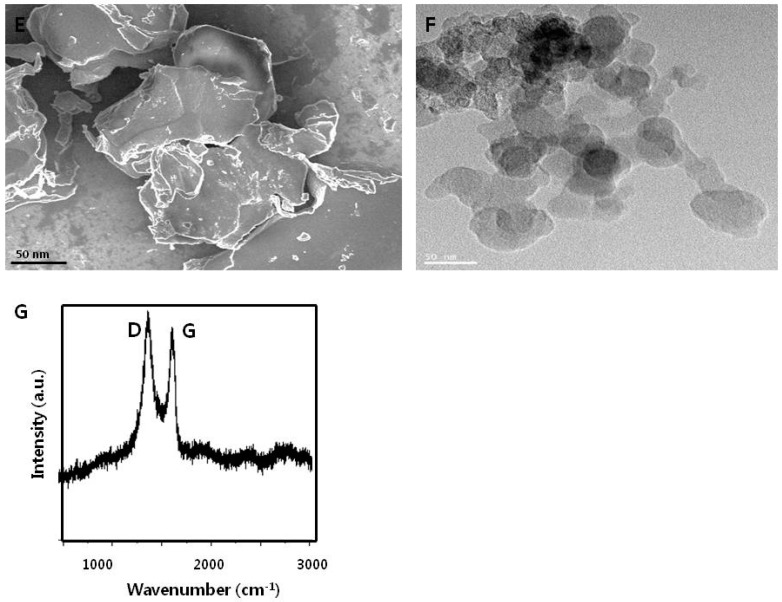
Synthesis and characterization of graphene oxide. (**A**) UV-visible spectrum of aqueous dispersions of graphene oxide. The absorption spectrum of graphene oxide exhibited a strong, broad peak at 230 nm. (**B**) FTIR spectrum of graphene oxide. (**C**) X-ray diffraction images of graphene oxide. (**D**) Dynamic light-scattering analysis of graphene oxide. (**E**) Scanning electron microscopy image of graphene oxide. (**F**) Transmission electron microscopy image of graphene oxide. (**G**) Raman spectrum of graphene oxide.

Graphene oxide materials were synthesized as described in our recent study [16], and then characterized using various analytical techniques, including ultraviolet-visible (UV-vis) spectroscopy (Biowave II spectrophotometer, Biochrom, Cambridge, UK). Fourier transform infrared (FTIR) spectroscopy (Perkin Elmer Inc., Waltham, MA, USA), X-ray diffraction (Bruker AXS GmbH, Karlsruhe, Germany), dynamic light scattering (Malvern Instruments, Malvern, UK), scanning electron microscopy (Tokyo, Japan), transmission electron microscopy (Tokyo, Japan), and Raman spectroscopy (Lise-Meitner-Str. Ulm, Germany). First, UV-vis spectroscopy was performed on the aqueous graphene oxide dispersions (Figure 1A)**.** Graphene oxide displayed two different characteristic features: A shoulder at approximately 300 nm, corresponding to an n-π* plasmon peak, and a separate shoulder at 230 nm, corresponding to a π-π* plasmon peak [32].

The FTIR spectra showed characteristic oxygen-containing groups in which the main absorption band at 3356 cm^−1^ was assigned to O–H group stretching vibrations. The absorption peaks at 1736 and 1625 cm^−1^ were assigned to C=O stretching of carboxylic and/or carbonyl functional groups [33]. The two absorption peaks at approximately 1365 and 1216 cm^−1^ were assigned to C–O stretching vibrations (Figure 1B). The FTIR results confirmed the existence of oxygen-containing groups on the graphene oxide nanosheets and the complete oxidation of natural graphene.

X-ray diffraction (XRD) was performed to determine the magnitude and location of peaks in the XRD curves. In general, natural graphite shows a sharp and highly intense reflection peak at 2θ = 26°. After oxidation, the sharp and intensive graphene oxide peak reflected at 2θ = 10.2° was due to the introduction of functional groups such as hydroxyl and carboxyl during oxidation and ultrasonic treatment (Figure 1C). This infers that the oxidation and ultrasonic treatment of natural graphite is effective for the preparation of graphene oxide [16,34].

Dynamic light scattering is a simple technique that can be used to determine the size distribution of particles in solution. Although the prepared sample had a wide particle size distribution (between 10 and 300 nm), most of the particles were dispersed within 50 nm (Figure 1D) in water and zeta potential was observed −28.30 mV. The size distribution analysis revealed that the prepared graphene oxide particles were 50 nm in size [17]. The size and zeta potential of graphene oxide was 90 nm and −15.0 mV (Table 1). The size was slightly larger and zeta potential highly negative. The prepared material was suitable to test the cytotoxicity nature in human cells.

The surface morphology of graphene oxide was confirmed by SEM. The SEM images indicate the formation of graphene oxide sheets with a closely packed lamellar structure, composed of waves that look like fine, alternating layers (Figure 1E). Graphene oxide exhibited an ultrathin, flexible, interconnected, sheet-like structure. A porous, network structure, like foam, was also observed due to extensive exfoliation of the graphene sheets [35]. Interestingly, graphene oxide sheets showed well-packed, overlapping curly layers. Furthermore, the surface morphology of the graphene sheets was formed by stacking of the exfoliated nanosheets [6]. Similarly, Yuan et al. [28] also observed that graphene oxide resembled a strongly folded, overlapping curtain rather than an aggregate.

Next, we examined the surface morphology of graphene oxide by TEM. The TEM images of graphene oxide exhibited a closely packed, lamellar, swollen structure, with a clean and transparent surface. The TEM images also showed a typical flaky structure, which had the wrinkly/wavy characteristic of transparent pure graphene oxide (Figure 1F). Our results are consistent with a previous report [36]. The interesting feature of this study is to produce such a small size of graphene oxide is very tedious. This study shows that exfoliation of graphite by sonication can be used to produce small-sized graphene oxide particles. Recently, Zhu et al. [22] observed that within prepared graphene nanosheets, approximately 75% of the sheets exhibited sizes between 100 and 300 nm.

Raman spectroscopy is used to observe the atomic vibrational, rotational, and other low-frequency dynamic modes in carbon materials. It is a very well designed technique to identify the molecular or atomic composition of samples quickly and reliably. As depicted in Figure 1F, Raman spectroscopy revealed a prominent Raman signal shift of the D band at approximately 1350 cm^−1^, which can be attributed to sp^3^-dependent defects on the breathing modes of the sp^2^ rings. These defects indicate flaws in the graphene structure and the G band at approximately 1600 cm^−1^, owing to the vibration of the sp^2^-bonded carbon atoms were observed in graphene oxide with the ID/IG value of 1.0. The D and G bands were attributed to disorders in the C–C bonds and in-plane vibrations of the C–C bonds, respectively, which is in agreement with previous reports [28,36,37,38].

### 3.2. Graphene Oxide Decreases the Viability and Proliferation of HEK293 Cells

**Figure 2:**
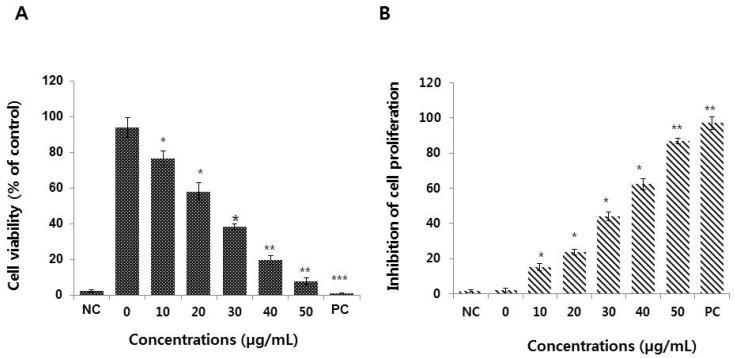
Effects of graphene oxide on the viability and proliferation of HEK293 cells. (**A**) The viability of HEK293 cells was determined by a CCK-8 assay after 24 h of exposure to different concentrations of graphene oxide. (**B**) Cell proliferation was assessed using a BrdU cell proliferation assay. The results are expressed as the means ± standard deviation of three independent experiments. A significant difference was observed between treated and untreated cells. *p < 0.05 was considered significant; **p < 0.01 was considered highly significant and ***p < 0.001 was considered very highly significant.

The effects of graphene oxide on the viability and proliferation of HEK293 cells were determined after 24 h of exposure to different concentrations of graphene oxide (0−50 μg/mL) using the CCK-8 and BrdU assays, respectively. The results revealed that graphene oxide has a significant and dose-dependent toxic effect, even at 10 μg/mL (Figure 2A,B). 

HEK293 cells show reduced (*p* < 0.05) viability and proliferation with increasing concentrations of graphene oxide. We have previously shown the dose-dependent toxic effects of graphene oxide in a variety of cells, including human breast cancer, ovarian cancer, neuroblastoma, and human lung cancer cells [17,28,31]. Recently, Zhu et al. [22] observed significant toxicity of graphene oxide in murine J774A.1 macrophages and human A549 lung cancer cells. Conversely, graphene oxide did not elicit significant cytotoxicity against J774A.1 and A549 cells at concentrations between 10 and 50 μg/mL [22]. Zhao et al. [39] demonstrated that graphene quantum dots (GQDs, <15 nm), small graphene oxides (SGOs, 50–200 nm), and large graphene oxides (LGOs, 0.5–3 μm) induced size-dependent cytotoxicity in murine macrophage-like Raw 264.7 cells. A human acute monocytic leukemia cell line treated with single- and multi-layered graphene oxide (SLGO and MLGO) (SLGO-15, -30 and MLGO-15, -30) and larger graphene oxides (SLGO-1, -5 and MLGO-1, -5) exhibited different levels of toxicity. Moreover, SLGOs are more cytotoxic than MLGOs. The degree of cell viability and proliferation was dependent on the size and concentration of the graphene oxide [40]. In accordance with previous findings, our study suggests that graphene oxide with an average size of 50 nm can induce cytotoxicity in HEK293 cells, even at low concentrations. Therefore, particle size is an important factor for the induction of toxicity.

### 3.3. Graphene Oxide Induces Cytotoxicity in HEK293 Cells

**Figure 3:**
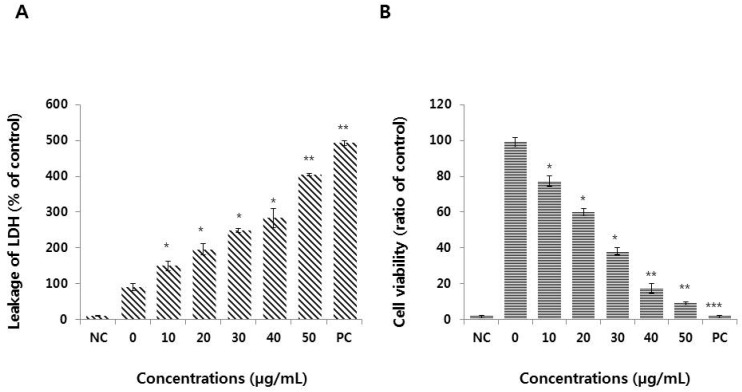
Measurement of lactate dehydrogenase (LDH) leakage and cell death-dependent protease activity in HEK293 cells. (**A**) LDH activity was measured at 490 nm using an LDH cytotoxicity kit. (**B**) The level of released dead-cell-dependent proteases was determined by the CytoTox-Glo cytotoxicity assay. The results are expressed as the means ± standard deviation of three independent experiments. There were significant differences in the levels of LDH and protease activity between graphene oxide-treated cells and untreated cells. *p < 0.05 was considered significant; **p < 0.01 was considered highly significant and ***p < 0.001 was considered very highly significant.

Although cytotoxicity can be measured by several cellular assays, methods that measure lactate dehydrogenase (LDH) leakage and dead-cell protease activity appear to be sensitive and reliable. Moreover, both assays have been used previously to measure plasma membrane integrity. Therefore, we measured LDH leakage and dead-cell protease activity to determine the cytotoxicity of graphene oxide. Dead-cell protease activity is represented as the percentage of viable cells. When the cell membrane is damaged, LDH is released into the medium, while dead-cell protease activity is quantified as the level of proteases released from membrane-compromised cells. HEK293 cells were treated with graphene oxide for 24 h and LDH and the dead-cell protease were dose-dependently released into the media (Figure 3A,B).

Graphene oxide induces cytotoxicity by increasing LDH leakage in a variety of cancer cells, including ovarian cancer, neuroblastoma, and human lung cancer cells [17,28,31]. Cho et al. [40] reported that the LDH level in SLGO-treated groups was higher than in MLGO-treated groups. The LDH level peaked at >40 μg/mL of SLGO and MLGO following a dose-dependent increase. In support of this mechanism of toxicity, Ding et al. [41] observed that GO-PEI (polyethyleneimine) -treated T lymphocytes exhibited compromised cell membranes due to the interaction between GO-PEIs and the cell membrane that eventually resulted in significant cytotoxicity. Recently, Zhu et al. [22] reported that graphene oxide, even at sub-lethal concentrations, can impair the general cellular priming state, including disrupting the plasma membrane and cytoskeleton construction, and inhibited several biological processes (e.g., morphological impairment) in J774A.1 macrophages and A549 lung cancer cells. These impairments of the plasma membrane eventually disrupt the cellular equilibrium of ions, proteins, and drugs, leading to cell injury, or even death [42]. Our results suggest that LDH leakage is dose-dependent and impairs the equilibrium state of the cells. Collectively, these findings suggest that the mechanism of membrane impairment and subsequent cytotoxicity is due to the physical interaction between cells and the nanomaterials [22,29,33,42,43].

### 3.4. Graphene Oxide Induces Oxidative Stress and Lipid Peroxidation in HEK293 Cells

**Figure 4:**
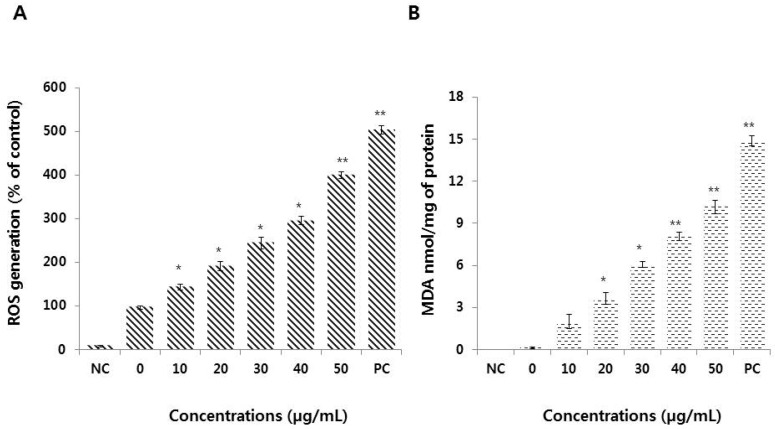
Measurement of reactive oxygen species (ROS) generation and malondialdehyde (MDA) levels in HEK293 cells. (**A**) HEK293 cells were treated with or without graphene oxide for 24 h and ROS generation was then measured using DCFH-DA. (**B**) HEK293 cells were treated with graphene oxide for 24 h. Lipid peroxidation was determined by the reaction of MDA with thiobarbituric acid (TBA) that forms a colorimetric (532 nm)/fluorometric (excitation and emission wavelengths of 532 nm and 553 nm, respectively) product, the quantity of which is proportional to the MDA present. The results are expressed as the means ± standard deviation of three independent experiments. There were significant differences in the levels of ROS generation and lipid peroxidation in graphene oxide-treated cells compared to untreated cells. *p < 0.05 was considered significant; **p < 0.01 was considered highly significant and ***p < 0.001 was considered very highly significant.

Oxidative stress is one of the major mechanisms involved in graphene oxide-induced oxidative stress in a variety of cell types, including bacterial and human cells [29,32,43]. Oxidative stress results from increased ROS levels that disrupt cellular homeostasis and affect the oxidation of biomolecules, including DNA, lipids, and proteins. We measured ROS generation in HEK293 cells using a DCFH-DA assay. Figure 4A shows that graphene oxide treatment resulted in a four-fold increase in ROS production compared to that in untreated cells.

Graphene oxide-treated HEK293 cells showed a high rate of ROS formation with increasing doses of graphene oxide (10–50 μg/mL). ROS combine with hydrogen atoms, generating water and a fatty acid radical that can cause oxidative degradation of lipids and, subsequently, cell membrane damage [43,44]. We examined the concentration-dependent production of malondialdehyde (MDA) in response to graphene oxide treatment. As shown in Figure 4B, graphene oxide induced lipid peroxidation (LPO) dose-dependently. Srikanth et al. [45] also demonstrated a dose-dependent increase in LPO in BF-2 cells with increasing concentrations of graphene oxide. Thus, our results are consistent with previous studies showing increased levels of ROS, MDA, and LPO in a variety of cells type, including human dental follicle, neuroblastoma, and human ovarian cancer cells, in response to graphene oxide treatment [46,47,48].

### 3.5. Effect of Graphene Oxide on Antioxidant Levels in HEK293 Cells

**Figure 5:**
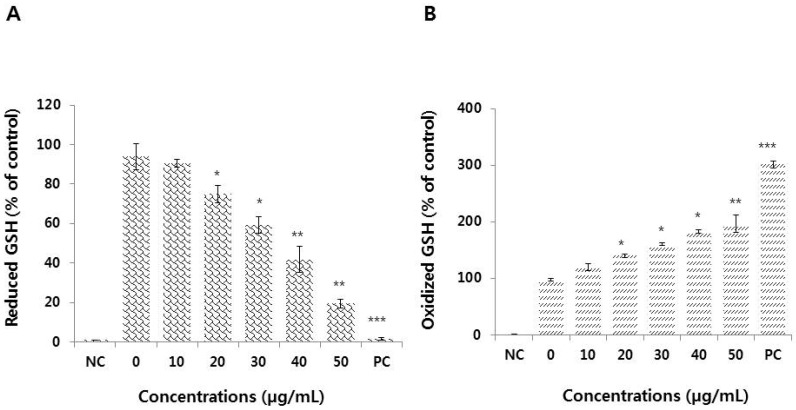
Measurement of antioxidant levels in HEK293 cells. Cells were treated with various concentrations of graphene oxide for 24 h. After incubation, the cells were harvested, washed twice with ice-cold PBS, and then disrupted by ultrasonication for 5 min on ice. (**A**) The concentration of glutathione (GSH) was expressed as mg/g of protein. (**B**) The concentration of oxidized glutathione (GSSG) was expressed as mg/g of protein. The results are expressed as means ± standard deviation of three independent experiments. Treated cells showed statistically significant differences compared to untreated cells. *p < 0.05 was considered significant; **p < 0.01 was considered highly significant and ***p < 0.001 was considered very highly significant.

Since thiol metabolism seems to play an important role in defense against exposure to toxicants such as nanoparticles, the concentration of reduced glutathione (GSH) was determined in HEK293 cells after incubation with graphene oxide. The level of GSH was significantly and dose-dependently decreased in graphene oxide-treated cells (Figure 5A). 

Glutathione is considered one of the most important ROS scavengers and is an important and ubiquitous component of the cellular defense mechanism against oxidative stress [49]. A rapid increase in ROS formation, along with decreased GSH levels, indicates that oxidative stress was the main cause of graphene oxide-induced toxicity. An inverse linear relationship between ROS and GSH levels was also observed with exposure to hybrid quantum dots, which decreased mitochondrial and cellular antioxidant levels [50].

Increased levels of oxidative stress disrupts homeostasis, and the enzymes responsible for reducing ROS levels, such as glutathione peroxidase (GPx), cannot compensate for the damage to macromolecules like proteins, DNA, and lipids, which greatly influences cell metabolism and signaling [9]. To better understand the mechanism of graphene oxide-induced toxicity, we further evaluated the oxidized glutathione level in graphene oxide-treated HEK293 cells. The results showed that graphene oxide treatment increased the level of oxidized glutathione level (Figure 5B). When cells are exposed to increased levels of oxidative stress by graphene oxide, the level of accumulation of GSSG could increase and the ratio of GSH to GSSG will decrease. The decreased level of reduced GSH and increased level of oxidized could be a possible mechanism by which graphene oxide creates an imbalance between the production of ROS and antioxidant levels, and the biological systems cannot withstand this unfavorable condition, which ultimately results in oxidative damage to various organelles.

### 3.6. Graphene Oxide Impairs Mitochondrial Function and Reduces ATP Generation

**Figure 6:**
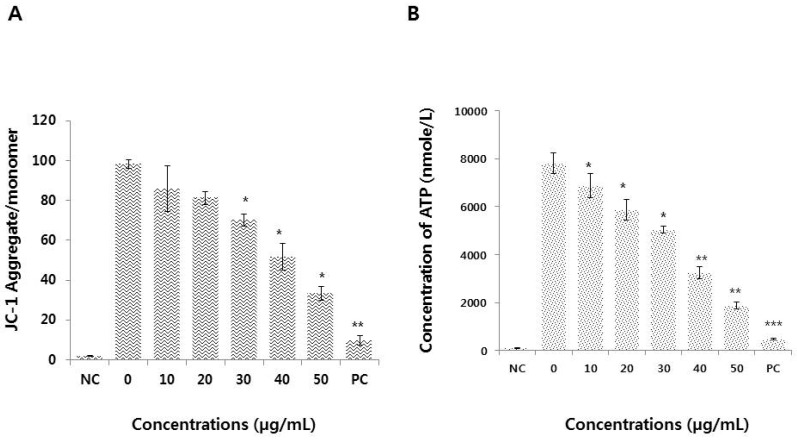
Measurement of MMP and ATP in HEK293 cells. (**A**) HEK293 cells were treated with various concentrations of graphene oxide for 24 h. Changes in MMP were then determined using a cationic fluorescent dye, JC-1. (**B**) ATP levels were measured according to the manufacturer’s instructions (Sigma-Aldrich; Catalog Number MAK135) in HEK293 cells exposed to graphene oxide for 24 h. The results are expressed as the means ± standard deviation of three independent experiments. There were significant differences in the ratios of MMP and ATP production in graphene oxide-treated cells compared to untreated cells. *p < 0.05 was considered significant; **p < 0.01 was considered highly significant and ***p < 0.001 was considered very highly significant.

Mitochondrial function is essential for cellular energy balance, metabolism, modulation of calcium signaling, and cell death. Since mitochondria are the main source of ROS in the cell, we hypothesized that a relationship exists between the mitochondrial membrane potential (MMP) and the rate of ROS production. Therefore, we investigated the effects of graphene oxide on the MMP by measuring the JC-1 aggregate to monomer ratio, and found that it was significantly and dose-dependently lower in graphene oxide-treated HEK293 cells than in the control condition (Figure 6A). The findings from this study demonstrate that graphene oxide has a pronounced effect on the MMP. Similarly, graphene quantum dots were found to elicit time- and concentration-dependent decreases in the MMP [51]. It is thought that graphene oxide enters the cytosol and mitochondria and that subsequently the internalized graphene oxide disrupts mitochondrial function [52]. Graphene oxide can disturb mitochondrial structure and function; as a result, the MMP is decreased, leading to dysregulation of mitochondrial Ca^2+^ homeostasis [14]. Loss of MMP is one of the crucial mechanisms by which mitochondrial dysfunction occurs, and overproduction of ROS ultimately results in mitochondrial-induced apoptosis [14,48].

Since mitochondria are a major source of intracellular ATP generation, and the MMP is vital for ATP production, we measured the ATP levels in graphene oxide-treated HEK293 cells. As shown in Figure 6B, graphene oxide exposure significantly decreased the ATP content in a concentration-dependent manner. The ATP reduction is indicative of a dysfunction in cellular metabolism. A previous study demonstrated that mitochondrial-produced ROS are a major cause of mitochondrial permeability transition (MPT), an important mechanism that induces apoptosis and necrosis [53]. Similarly, graphene oxide quantum dots can cause significant toxicity in human lymphocytes due to mitochondrial dysfunction [50]. Exposure of MDA-MB-231 human breast cancer, PC3 human prostate cancer, and B16F10 mouse melanoma cells to graphene leads to the direct inhibition of electron transport chain (ETC) complexes I, II, III, and IV, resulting in mitochondrial depolarization and impaired ATP production [35].

### 3.7. Graphene Oxide Activates Caspase 3 and Induces DNA Damage in HEK293 Cells

**Figure 7:**
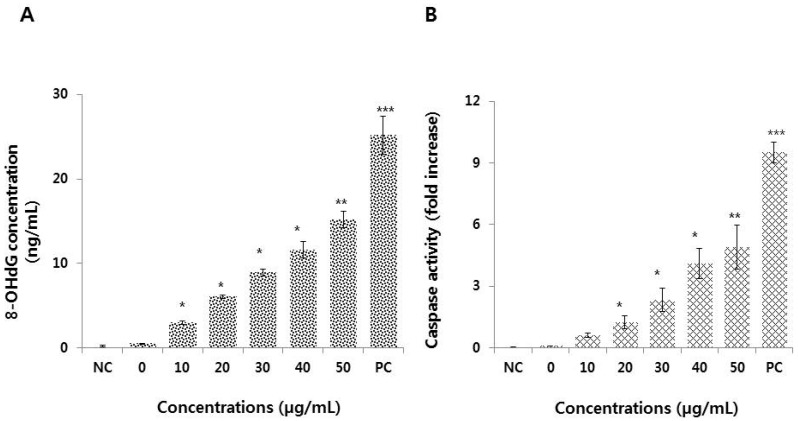
Graphene oxide induces DNA damage and caspase 3 activity in HEK293 cells. (**A**) Cells were treated with various concentration of graphene oxide for 24 h. DNA damage was estimated by ELISA based on the levels of 8-oxo-dG. (**B**) Caspase 3 activity was measured based on the concentration of p-nitroanilide released from the substrate, which was calculated from the absorbance at 405 nm. Results are expressed as the means ± standard deviation of three independent experiments. The treated groups showed statistically significant differences from the control group. *p < 0.05 was considered significant; **p < 0.01 was considered highly significant and ***p < 0.001 was considered very highly significant.

Overproduction of ROS can induce oxidative stress, which can subject cells to DNA damage, unregulated signaling, changes in motility, cytotoxicity, apoptosis, and cancer initiation [54]. DNA damage can be measured using typical biomarkers like 8-hydroxy-20-deoxyguanosine (8-OH-dG). To investigate whether graphene oxide exposure results in DNA damage, cells were exposed to various concentrations of graphene oxide for 24 h. The cell lysates were obtained by collecting the supernatant of the mixture after centrifugation and the levels of 8-OH-dG were then measured. Graphene oxide treatment showed dose-dependent effects on 8-OH-dG concentrations, whereas untreated cells showed no significant levels of 8-OH-dG (Figure 7A). Under certain threshold conditions, genomic instability can be attenuated through DNA replication-coupled repair mechanisms, whereas more sensitive cells, such as germ and stem cells, may be redirected to cell cycle arrest and apoptosis [55]. However, the molecular mechanisms of genotoxicity and cytotoxicity remain unclear. This study demonstrated a correlation between DNA damage and cytotoxicity, where exposure to 25 µg/mL graphene oxide resulted in 80% cell death. Our study agrees with a previous study where exposure of human mesenchymal stem cells to reduced graphene oxide nanoparticles induced DNA fragmentation and chromosomal aberrations by penetrating and eventually killing the cells [56]. Similarly, Zhang et al. [57] reported that a reduced graphene oxide-silver nanocomposite induced DNA damage in human ovarian cancer cells by increasing the expression of RAD51 and phosphorylation of H2AX. Overall, oxidative DNA damage involves base and sugar lesions, DNA-protein crosslinks, single- and double-strand breaks, and formation of abasic sites [58]. Collectively, the results show that graphene oxide can induce cell death through DNA damage.

Overall, caspases play significant roles in cell survival and cell death, since most apoptotic pathways are activated by caspase-dependent cleavage of numerous cytoplasmic and nuclear proteins. Apoptotic signaling by executioner caspases, including caspases 3, 6, and 7, is activated by initiator caspases (e.g., caspase 2, 8, 9, and 10) [59,60]. To investigate the involvement of caspase 3 in DNA damage, we estimated the level of caspase 3 activity. The results indicated that graphene oxide dose-dependently increased the activity of caspase 3 in HEK293 cells (Figure 7B). A previous study suggested that silver nanoparticles (AgNPs) reduced cell viability by the formation of apoptotic bodies, sub-G1 hypodiploid cells, and DNA fragmentation through increased oxidative stress, leading to cytotoxicity and apoptosis [61]. Roos and Kaina (2006) reported that DNA damage activates caspases through p53 in both *C. elegans* germ cells and mammalian cells [62]. The p53 protein is a downstream target of DNA damage and activates various pro-apoptotic genes, including *PUMA* and *NOXA*. Graphene nanoparticles induce cytotoxicity in rat pheochromocytoma cells (PC12 cells) through mitochondrial injury-dependent release of LDH, increased activation of caspase 3, and ROS generation [10]. Orth et al. [63] demonstrated that the DNA damage response (DDR) can be induced by mitotic arrest, which is a consequence of caspase activation, and demonstrated that the mitotic DDR at telomeres depends on sub-apoptotic activation of the classical caspase 9/3/7 pathway under the control of MCL1 and other BCL2 family proteins. Recently, Lu et al. [64] observed a significant decrease in cell viability and increased DNA damage in HEK293T cells at high graphene oxide doses (25 and 50 mg/mL). Our findings revealed reduced cell viability and proliferation, and increased oxidative stress and DNA damage, even with low graphene oxide concentrations (5 and 10 mg/mL), likely due to the small size of the particles. Collectively, this study suggests that the crosstalk between DNA damage and caspase 3-mediated induction of apoptosis in HEK293 cells is dependent on the dose of the graphene oxide.

### 3.8. Graphene Oxide Treatment Induces Changes in Gene Expression

**Figure 8:**
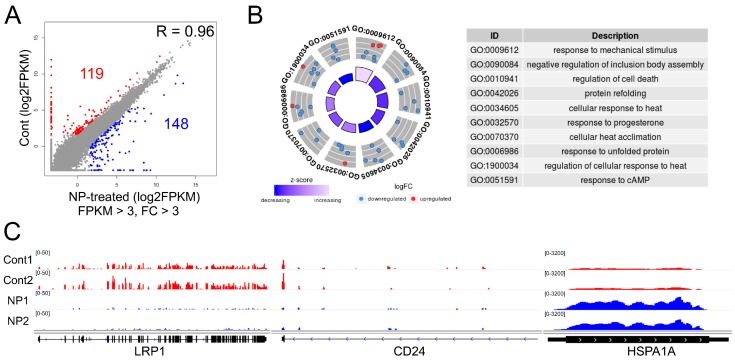
Graphene oxide alters gene expression patterns. (**A**) Scatterplot showing differentially expressed genes (DEGs) in RGO-exposed HEK293 cells. Red and blue dots represent genes respectively down- and upregulated in the experimental group. Log_2_FPKM values were used for plotting. Cutoff values: FPKM > 3 and fold change (FC) > 3. (**B**) GO term analysis of the DEGs. (**C**) IGV browser images of representative down- (*LRP1* and *CD24*) and upregulated (*HSPA1A*) genes.

Next, we analyzed the mechanism by which graphene oxide exposure affects cell survival, we produced more than 20 million RNA-Seq reads from each sample of HEK293 cells (two samples/group). In the DEG analysis, 119 and 148 genes were down- and upregulated, respectively, in response to graphene oxide treatment (Figure 8A). We next analyzed the biological pathways that are dysregulated by graphene oxide exposure. As shown in Figure 8B, using 267 whole DEGs, multiple GO terms were obtained from the DAVID analysis, including response to mechanical stimuli, negative regulation of inclusion body assembly, and regulation of cell death. Some representative down- (*LRP1* and *CD24*) and upregulated genes (*HSPA1A*) are illustrated using the IGV genome browser (Figure 8C, Supplementary Table S1 and S2).

### 3.9. Cell Survival-Related Genes Were Affected by Graphene Oxide

**Figure 9:**
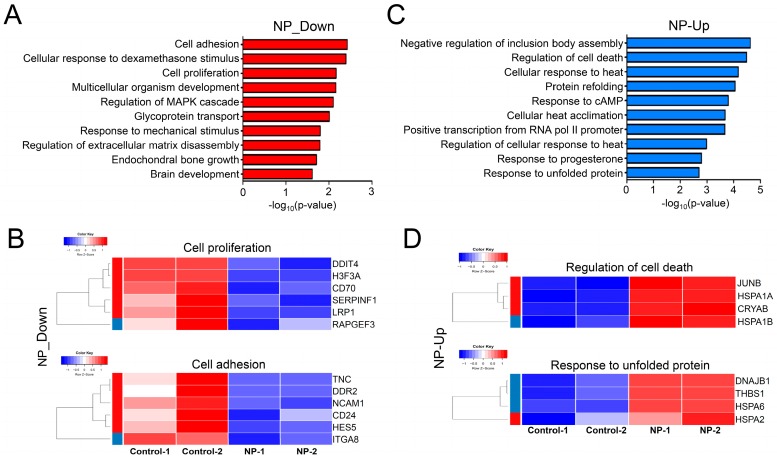
Analysis of gene ontology for the down- or upregulated genes. (**A**) Bar graphs displaying the top 10 GO terms in the genes repressed by graphene oxide. The *x*-axis scale represents -log_10_(*p*-values) values. (**B**) Heatmaps showing the expression of genes in the GO terms of cell proliferation and cell adhesion. NP, nanoparticle. (**C**) Bar graphs displaying the top 10 GO terms in the genes enhanced by graphene oxide. (**D**) Heatmaps showing the expression of genes in the GO terms of regulation of cell death and response to unfolded protein.

We then conducted separate GO term analyses of down- or upregulated genes. Genes related to cell adhesion and proliferation were repressed, as shown in Figure 9A. In agreement with this, Park et al. [65] showed that repression of *DDR2*, a gene found in the GO term of “cell adhesion” (Figure 9B), inhibited tumor cell proliferation both in vivo and in vitro. In gastric cancer cells, depletion of *CD24*, a gene found in the GO term of “cell adhesion”, resulted in inhibition of tumor metastasis and increased apoptosis and chemosensitivity to doxorubicin [66]. Inactivation of *LRP1*, a gene in the GO term of “cell proliferation“, was shown to increase apoptosis by activating the NF-κB pathway [67]. Furthermore, Zhu et al. [22] demonstrated that exposing cells to graphene oxide, including A549 lung cancer cells and J774A.1 macrophages, led to impaired expression of focal adhesion and cytoskeleton proteins. The expression of representative genes in the GO terms of “cell proliferation” and “cell adhesion” is shown in heatmaps in Figure 9B.

In contrast, GO term analysis of the upregulated genes revealed that biological pathways, including “negative regulation of inclusion body assembly“ and “cell death“, were significantly altered by graphene oxide treatment (Figure 9C). Previously, the expression of *HSPA1A*, a gene in the “negative regulation of inclusion body assembly” GO term (Figure 9), was shown to be enhanced in response to 6-hydroxy-2,2′,4,4′-tetrabromodiphenyl ether (6-OH-BDE-47) exposure [68]. Moreover, improper formation and accumulation of inclusion bodies were reported to elicit harmful effects on cell function and viability [69]. Phukan et al. [70] demonstrated that exposing HEK293 cells to silica-coated magnetic nanoparticles aggravated proteasome function by reducing the expression of its regulators such as PSMA1, PSMA7, and PSME1 and increasing inclusion body assembly in the cytoplasm. In addition, GO terms associated with the “mechanism of protein folding” were also detected in the analysis. The expression of representative genes in the GO terms of “regulation of cell death” and “response to unfolded protein” is shown in heatmaps in Figure 9D.

### 3.10. Apoptosis-Related Pathways Were Aberrantly Activated by Graphene Oxide

**Figure 10:**
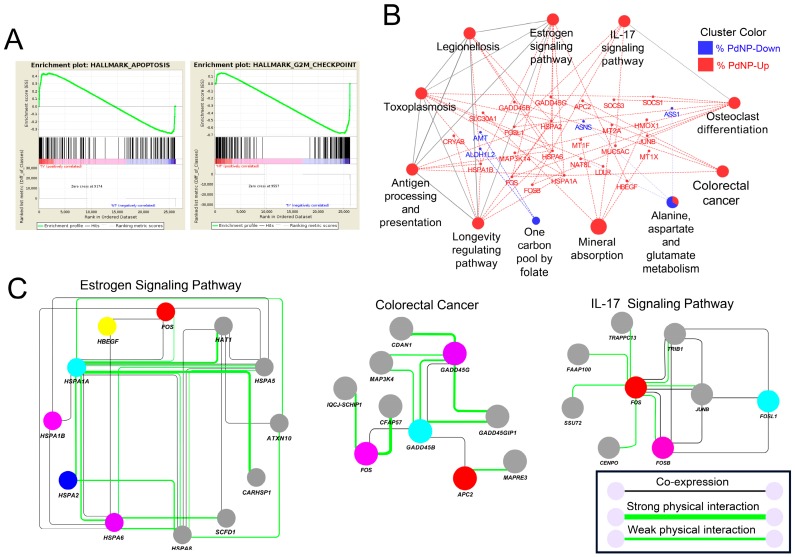
Apoptosis-related pathways were significantly altered by graphene oxide. (**A**) Gene set enrichment analysis (GSEA) showing enriched pathways in the DEGs. Note that genes in the apoptosis and G2/M checkpoint pathways were significantly changed. (**B**) KEGG analysis showing gene networks and pathways altered by graphene oxide. Red color: Upregulated genes; blue color: downregulated genes. (**C**) Networks showing coexpression and protein-protein interactions in the estrogen, IL-17, and colorectal cancer signaling pathways.

Next, analysis of pathways using gene set enrichment analysis (GSEA), KEGG, and GeneMANIA was performed to know the mechanism by which graphene oxide promotes apoptosis. In the GSEA, 33 out of 50 and 17 out of 50 gene sets were respectively up- and downregulated in the graphene oxide-treated group, with an enrichment score of 0.4. Among them, the analysis revealed that groups of DEGs were enriched in the apoptosis and G2M checkpoint pathways (Figure 10A). KEGG analysis showed that pathways closely linked to apoptosis were altered by graphene oxide exposure (Figure 10B), including the estrogen, IL-17, and colorectal cancer signaling pathways, among others. Further analysis uncovered that some proteins in the pathways interact physically and are also coexpressed in the same cells (Figure 10C). Therefore, our analysis indicates that exposing HEK293 cells to graphene oxide elicits apoptosis directly by changing gene expression.

### 3.11. Altered Expression of Key Transcription Factors Promotes Apoptosis by Altering Target Gene Expression 

**Figure 11:**
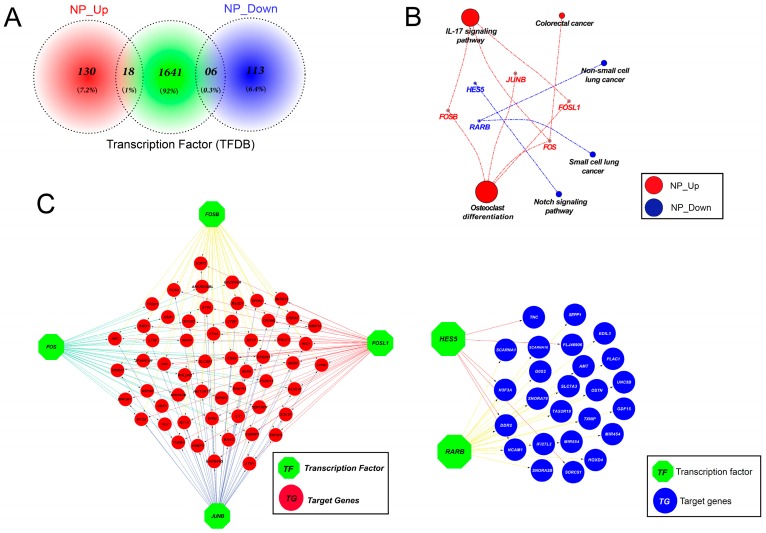
Graphene oxide altered transcription factor (TF) expression, resulting in changes to downstream gene expression. (**A**) Venn diagram showing TFs among the DEGs. Eighteen out of 148 upregulated genes and six out of 119 downregulated genes were identified as TFs. (**B**) KEGG analysis with TFs differentially expressed following graphene oxide exposure. Red color: Upregulated genes; blue color: Downregulated genes. (**C**) Networks showing differentially expressed target genes downstream of the TFs identified in (**A**).

To study the precise mechanism by which the nanoparticles impair gene expression, transcription factors (TFs) were queried among the DEGs. As shown in Figure 11A, 18 and six TFs were found among the up- and downregulated genes, respectively, and the identified TFs were subjected to KEGG pathway analysis. Genes coding for transcription factors in the IL-17, colorectal cancer, and osteoclast differentiation signaling pathways were upregulated, including *FOSB*, *JUNB*, *FOS*, and *FOSL1*. Irradiating mammalian cells with ultraviolet (UV) light is known to rapidly induce *c-Jun*, *JUNB*, and *c-Fos* expression [71]. In contrast, TFs in non-small cell lung cancer, small cell lung cancer, and the Notch signaling pathway were downregulated. These TF-encoding genes include *HES5* and *RARB* (Figure 11B). Importantly, 55 out of 119 upregulated genes and 25 out of 148 downregulated genes were identified as direct downstream targets of the upregulated and downregulated TFs, respectively (Figure 11C). Collectively, our analysis not only establishes mechanistic insights into how graphene oxide exposure greatly induces apoptosis in HEK293 cells, but also provides a basis for the development of therapeutic strategies to ameliorate harmful effects during use. Follow-up studies are needed to determine whether exposing other cell types to different nanoparticles induces apoptosis by the same or a similar mechanism.

## 4. Conclusions

Understanding the molecular mechanism of graphene oxide-induced cytotoxicity and cellular responses in HEK293 cells is necessary. We therefore proposed an integrative analytical approach, using various cellular assays and transcriptome analysis, to identify the associated molecular and cellular pathways. We prepared smaller graphene nanoparticles with an average size of 50 nm and then characterized them using various analytical techniques. Furthermore, we also performed a series of assays that measured cell viability, proliferation, cytotoxicity, oxidative stress, antioxidant levels, mitochondrial dysfunction, DNA damage, caspase 3 activity, and the transcriptional profiles of genes involved in various cellular pathways. The CCK-8 and BrdU assays revealed a significant, dose-dependent decrease in cell viability and proliferation in response to graphene oxide. Our findings also demonstrated increased LDH leakage, ROS generation, and decreased levels of glutathione and glutathione peroxidase. This detailed, mechanistic approach shows that graphene oxide exposure results in decreased mitochondrial membrane potential and ATP synthesis, as well as increased DNA damage and caspase 3 activity. Our analysis revealed that graphene oxide directly alters the expression of genes involved in various apoptosis-related biological pathways. The RNA-Seq analysis of graphene oxide-treated HEK293 cells not only establishes mechanistic insights into how graphene oxide evokes extensive cell death in HEK293 cells, but also provides a basis for the development of therapeutic strategies to alleviate harmful effects during use. This study further provides a transcriptomic insight into the role of surface-mediated cellular signaling triggered by graphene oxide and will enable the development of nanomaterial-based therapeutics for regenerative medicine. This approach in understanding nanomaterial–cell interactions illustrates how changes in the transcriptomic profile can predict downstream effects following nanomaterial treatment.

## Figures and Tables

**Table 1:** Dynamic light scattering analysis and zeta potential measurement of graphene oxide in water and culture media.

Sample	Hydrodynamic Size in Water (d.nm)	Hydrodynamic Size in Media (d.nm)	Zeta Potential in Water (mV)	Zeta Potential in Water in Media (mV)
Graphene oxide	50	90	−28.30	−15.0

## Supplementary Materials

The following are available online at https://www.mdpi.com/2079-4991/9/7/969/s1, Table S1: HEK293-Treatment-Up-Regulated-Genes; Table S2: HEK293-Treatment-Down-Regulated-Genes.
