# Supplementary material for: Evaluation of Graphene Oxide Induced Cellular Toxicity and Transcriptome Analysis in Human Embryonic Kidney Cells
Source: Nanomaterials (Basel). 2019 Jul 2;9(7):969. doi: 10.3390/nano9070969 (PMC6669460; doi:10.3390/nano9070969)
Supplement: Supplementary file 1 [file nanomaterials-09-00969-s001.pdf]

Supplementary Table S1. HEK293-Treatment-Up-Regulated-Genes.

Symbol	FPKM (Control1)	FPKM (Control2)	FPKM (Treatment1)	FPKM (Treatment2)	Log2FC
ABHD11-AS1	0	0	4.45561	3.9924	5.434296
ACRC	1.31082	1.03398	10.7299	9.41993	2.999393
ACTN2	2.44821	2.51843	8.40331	7.5381	1.643468
ANKRD30BL	415.575	413.05	9976.04	11987.9	4.727939
ANXA6	31.2692	22.3066	92.9458	106.506	1.892457
APC2	0.224074	0.174551	4.99473	5.10898	4.105368
ARC	1.34272	1.61215	137.544	141	6.465216
ASB1	6.9858	7.45154	20.7373	26.7161	1.702926
ATN1	6.55978	6.73679	24.609	29.5317	2.00944
BAG3	7.90873	10.5781	41.4866	36.9555	2.07328
C19orf26	1.91453	1.92258	4.30066	8.22943	1.656848
C9orf173	0.120528	0	3.08617	8.30289	5.176169
CAPN15	20.2166	13.4925	53.4824	54.2645	1.670577
CCDC86	32.7102	24.8082	86.8066	93.1418	1.642083
CD68	0.948763	2.36054	10.5745	7.7819	2.40266
CDKN1C	1.82948	2.09094	14.3732	13.914	2.789451
CHGA	0.91083	2.12501	7.32445	7.00746	2.167012
CLU	2.95305	7.88171	29.4788	24.8029	2.303716
CORO7-PAM16	7.74E-06	0.94293	3.3485	5.27124	2.947989
CRYAB	0.137821	0.160316	14.1008	15.8047	5.917341
CSRNP1	3.14259	6.01844	15.1315	13.0627	1.600857
CTGF	1.32456	0.801408	7.10536	6.04271	2.520728
DHRS2	3.25791	1.98184	19.7983	20.6822	2.902725
DIAPH1	28.7895	27.4294	78.7691	97.4412	1.644684
DIO3	0.165558	0.386278	3.90578	3.8217	3.398372
DLL1	4.85297	6.22053	29.0893	27.5973	2.335153
DNAH17	0.345726	1.30387	3.27294	3.3989	1.893486
DNAJB1	19.8567	28.334	197.99	179.19	2.963216
DRD4	0.895232	0.730155	3.73397	2.74766	1.871998
EEF1E1-BLOC1S5	1.68833	0.00257302	0.297226	6.62929	1.914122
EN2	5.78229	6.09521	17.7823	19.1028	1.618518
EPPK1	7.28904	8.57976	36.9564	40.7908	2.278235
ETV4	0.306192	0.353574	2.94809	4.0747	3.07054
ETV5	0.54193	0.949287	4.54286	3.65709	2.312319
FABP3	0.11519	0	3.31955	3.52803	4.482834
FAM222A	1.75818	0.837764	4.73985	4.9775	1.826619
FOS	18.8239	14.0151	62.6352	52.6202	1.805094
FOSB	5.76209	3.10528	30.0781	33.328	2.810409
FOSL1	0.269692	0.70904	9.31293	7.07018	3.814407
GADD45B	10.1838	11.247	175.885	149.159	3.910364
GADD45G	0.477282	1.94427	11.8403	11.8733	3.189338
GLTSCR1	1.37719	1.49701	4.07277	6.42125	1.798521

HBEGF	2.24117	2.95626	9.87949	7.3142	1.688219
HIC1	0.499483	0.624749	4.59812	5.98357	3.025356
HIST1H1T	1.34942	0.671325	4.29492	2.93362	1.742035
HMOX1	21.0341	51.8436	118.958	120.035	1.710672
HSPA1A	0.330315	0.495588	71.3934	65.7627	7.064883
HSPA1B	29.3923	45.3115	358.949	332.88	3.207722
HSPA2	0.984202	2.15392	4.22632	6.31819	1.686491
HSPA6	0.0974421	0.113702	372.441	312.448	10.70243
HSPA7	0.0458864	0.107098	12.1135	10.0704	5.986713
IGF2-AS	0.096541	0.264356	9.52037	6.53797	4.8573
IL11	0.104772	0.281685	4.35121	3.71699	3.817476
INO80B-WBP1	0.671944	0.00331386	15.0271	12.8429	5.003177
JOSD2	5.37202	2.17353	10.1946	14.564	1.688097
JUNB	6.80299	6.57355	26.0347	24.9072	1.91339
KCNC3	1.70398	0.80992	4.47463	5.46163	1.901086
KDM6B	2.88537	2.70427	10.468	13.0238	2.032842
KLHL21	14.267	8.78923	46.4031	40.0886	1.898277
LDLR	2.37513	1.19805	8.80399	8.03824	2.175261
LFNG	0.677681	0.856423	3.73903	2.9645	1.993144
LINC00910	2.14419	2.09805	13.5301	17.6859	2.82214
LOC649352	0	0.351329	22.0537	22.9147	6.356257
LY6K	0	0.247363	3.05724	5.05683	4.216037
MAFA	0.629056	1.41161	9.18938	12.6664	3.299157
MAFA-AS1	0	0.501067	2.71989	6.49759	3.747717
MAFF	0.571213	1.02984	13.7628	10.9905	3.792318
MAP3K14	1.58015	1.32641	5.73253	6.38021	1.986762
MIDN	7.35231	8.07009	25.881	26.7589	1.758012
MIR10A	0	0	267.469	0	10.38623
MIR22HG	0.471999	1.45645	3.38602	5.25103	2.053762
MIR3648-1	87.9799	182.84	1926.5	2112.96	3.897763
MIR5047	0	0	694.486	0	11.76215
MIR635	0	0	863.338	0	12.07604
MIR658	0	0	0	886.363	12.11401
MIR663A	0	808.989	3135.88	8044.55	3.788381
MIR663AHG	34.4904	35.4001	153.194	168.663	2.200025
MIR663B	0	81.3144	178.038	222.379	2.297097
MIR6753	0	0	16.6715	0	6.398444
MIR7-3HG	0.3577	0	9.1177	8.85558	5.026186
MMP10	0	0	5.39305	3.86959	5.564171
MROH6	0.96876	0.646284	2.67563	3.86673	1.893249
MT1F	3.17255	2.74198	37.2718	40.0717	3.664692
MT1X	13.3321	8.45862	384.135	381.768	5.122572
MT2A	28.5028	16.9005	646.321	655.966	4.835987
MTRNR2L2	0.616644	0.287042	38.2811	43.8823	6.221602
MTRNR2L8	0	0	20.7808	25.2139	7.851584
MTRNR2L9	0.300449	0.694818	91.0363	98.5822	7.311144

MUC5AC	0	0	3.04236	3.63886	5.104592
NAT8L	10.8744	7.81379	30.9243	34.9687	1.807013
NEFH	0.0300229	0.0701132	6.48396	7.17621	5.529184
NRARP	9.61071	8.83116	28.0058	27.7999	1.587027
NUDT4P2	0.00279547	0.0032949	5.77069	8.56514	6.140193
OSGIN1	0.240978	0.702682	3.29346	3.24356	2.558452
PARD6A	2.64388	1.02602	5.33629	7.13255	1.710916
PCDH17	2.35052	0.96833	5.52536	5.1402	1.626587
PHLDA2	1.28225	4.30779	22.7616	21.7699	2.949646
PHOSPHO2-KLHL23	0.918057	0.000334926	3.31689	6.05732	3.097728
PLIN4	0.292865	0.523252	2.94607	3.05832	2.610223
POU3F1	0.38642	0.496173	2.98738	3.57285	2.642581
PRR35	0.468733	1.033	4.18973	3.60763	2.232519
PRX	1.22719	0.743316	4.50303	5.61766	2.249436
PTOV1-AS2	7.56976	4.0054	18.7305	21.0778	1.764553
PYCARD-AS1	0.808076	0.671891	4.80521	2.65171	2.188331
RASD1	0.213024	0.315647	13.7022	14.8253	5.301021
RFPL4A	0.289595	0.168374	11.5644	2.76513	4.464825
RND1	1.855	1.41412	5.04316	5.48256	1.628433
RNU11	715.96	1073.83	6796.88	6507.02	2.893847
RPL13P5	2.595	0.54684	4.49005	6.26069	1.712314
RPL36A-HNRNPH2	0.00167956	0.00960572	9.58463	3.27892	5.950211
RPS10-NUDT3	0.933663	0.763698	5.86422	3.84807	2.385224
RRAD	0	0	35.0705	34.9794	8.456352
RTEL1-TNFRSF6B	2.51935	0.134882	2.59687	6.85674	1.757966
SCRT1	0.0295316	0.103451	2.85587	3.30182	4.254984
SERTAD1	5.67812	4.32823	36.3967	35.2114	2.81468
SETD1B	5.18927	3.96963	14.5216	15.4221	1.687446
SGK1	3.8867	2.83594	12.7872	9.35578	1.690427
SLC30A1	8.25081	8.0092	31.7343	24.4129	1.775379
SLC9A1	3.26229	2.33285	9.54388	10.6062	1.812119
SNAI1	1.11102	1.13351	20.975	19.7461	4.065216
SNORA36A	0	0	80.0375	0	8.648133
SNORA51	0	0	59.3027	145.303	10.00004
SNORA55	17.9321	0	45.9433	56.0408	2.494553
SNORA56	0	0	5.48E-09	118.755	9.2162
SNORA66	22.1525	0	0	206.484	3.208919
SNORA77	0	0	88.955	0	8.800172
SNORA80E	0	0	53.3779	195.817	10.28422
SNORD83A	0	0	1222.27	0	12.57751
SOCS1	0.306153	0.475403	2.94393	4.30233	2.923374
SOCS3	0.373681	1.11461	2.80802	3.3583	1.914896
SOX8	1.73977	2.37664	10.9713	13.8346	2.534365
SPANXN3	0	0	5.65655	6.76002	5.979176

SPHK1	3.34424	4.0164	13.5252	15.0677	1.929133
TFPI2	0.703145	1.81529	5.43245	3.97034	1.820677
THBS1	0.105412	0.33427	5.92889	5.6964	4.208377
TNFSF9	3.21058	5.32376	14.9002	14.5288	1.762239
TOR4A	1.17502	1.2126	4.40505	5.26857	1.931953
TRIM47	1.12058	1.1886	3.27832	4.31269	1.634594
TUBB3	5.60263	3.40559	31.9553	29.7532	2.749143
USB1	4.76495	4.26949	16.0762	19.5542	1.956088
USP17L2	0	0.0873891	4.33417	7.19848	5.35138
USP17L7	0	0	6.98283	4.03115	5.809155
VASN	0.485187	0.7079	8.46447	6.69694	3.462953
VGF	0.492027	0.626416	13.6815	15.329	4.46958
ZFAND2A	11.2566	8.83916	43.4285	35.1715	1.957017
ZFP36	0.943823	1.80809	14.3004	11.9191	3.161881
ZNF296	1.13001	1.14167	6.53326	10.7062	2.818791
ZNF778	8.12134	7.96139	27.2739	35.7184	1.956406

Supplementary Table S2. HEK293-Treatment-Down-Regulated-Genes.

Symbol	FPKM (Control1)	FPKM (Control2)	FPKM (Treatment1)	FPKM (Treatment2)	Log2FC
ABCG1	3.00545	7.91237	0.850696	1.32484	-2.226548955
ACTA2	7.2938	15.7981	2.78434	1.29067	-2.44582863
AGAP11	1.99924	5.2514	0.805183	1.23813	-1.731733429
ALDH1L2	13.1125	23.1896	5.45769	5.35621	-1.728654051
AMT	3.6503	8.02603	0.99094	0.689177	-2.659194737
AMY2B	2.77842	3.90209	1.49015	0	-2.025364214
ARRDC3-AS1	3.19963	3.30723	0.735787	1.05596	-1.751603085
ASNS	179.335	409.171	100.612	86.5299	-1.651873729
ASS1	31.1922	100.984	12.0914	13.6987	-2.348609673
C6orf48	202.14	437.521	73.7694	69.4496	-2.157522372
CALCB	32.6244	125.154	15.0833	15.6609	-2.351986386
CD24	2.79232	6.78612	1.12253	1.79127	-1.650928041
CD70	4.60223	5.51901	1.37847	1.648	-1.677587791
CDC37L1-AS1	6.15765	12.3745	0	4.94064	-1.865496607
CHAC1	33.9218	41.0631	11.4608	10.6109	-1.755231259
CNPY1	3.46068	5.90594	0.66485	1.59022	-1.962245134
COL14A1	6.04166	7.11728	1.69281	1.37159	-2.032915785
DDIT4	124.315	135.607	45.2847	39.3847	-1.615872038
DDR2	4.34238	13.9586	2.17894	2.0328	-2.068182017
DICER1-AS1	6.08107	5.88756	2.69314	0	-2.072458709
DMC1	17.3212	34.3154	7.81834	8.58121	-1.642826962
EBF4	2.91614	7.76105	1.87867	1.19832	-1.730862725
EDA2R	5.18755	4.93213	0.84375	1.00913	-2.329677032
EDIL3	1.93638	5.05598	1.19767	0.901034	-1.645644529

ELSPBP1	1.92205	16.3581	0.76901	0.459727	-3.69316419
FGF21	1.94315	8.34151	0	0	-5.712136268
FLJ46906	12.7255	23.4805	2.87812	3.93266	-2.376529369
FUT1	2.35247	11.508	1.09565	1.00802	-2.609638849
G0S2	9.56516	52.8124	2.58434	5.1496	-2.979536014
GDAP1L1	0.775167	8.10129	0.138654	0.362985	-3.693328405
GDF15	3.8347	21.4837	0.664314	0	-4.883838535
GS1-259H13.2	1.68946	6.3337	1.45147	0	-2.315942138
H3F3A	5.43512	6.3259	0.00735355	0.00875294	-5.790454214
HES5	2.72446	6.56667	0.903953	1.0809	-2.119043646
HHIP	2.99047	6.21151	0.507282	1.09209	-2.385471133
HOXD4	11.374	5.52077	1.5199	3.63482	-1.674671969
HSPB8	3.14294	18.3986	2.24636	2.23868	-2.214320863
IFI27L2	12.2948	24.1987	4.54375	5.42839	-1.850901299
INHBE	5.12531	32.2093	0.302732	0.362051	-5.43973952
ITGA8	4.29958	3.51823	0.9165	1.21802	-1.780082282
JDP2	2.92422	4.79972	1.04688	0.870853	-1.903697248
KRTAP19-1	46.308	170.551	18.5518	18.4624	-2.544162115
LINC01021	6.61657	6.64004	0	0.436791	-4.401351255
LINC01411	2.11459	4.92265	0.752195	1.34904	-1.653031297
LINC01535	4.60417	6.33535	1.37663	0.928744	-2.15258918
LINC01604	13.0269	9.62793	3.40966	3.8669	-1.612052551
LOC105373383	5.02545	3.23078	0.882662	1.05477	-1.984136094
LRP1	7.0617	12.421	2.78033	2.9064	-1.741389774
MIR1291	1800.93	0	0	0	-13.13661469
MIR454	74.1865	0	0	0	-8.538897008
MIR503HG	8.15924	3.8315	1.10671	1.32297	-2.212826546
MIR554	0	549.06	0	0	-11.42327352
MIR7844	32.2596	0	0	0	-7.342501411
NCAM1	8.99398	14.0122	3.85513	3.45959	-1.626717803
NT5M	2.03115	4.20946	0.966531	0.866851	-1.663316063
NUDT4P1	4.48963	5.923	0.419937	0.459401	-3.297563599
NUP50-AS1	4.58769	5.6418	1.75861	1.05144	-1.792809255
NUPR1	11.5465	53.2439	2.4796	2.96432	-3.525457184
OSTN	4.63713	4.26544	0	1.72628	-2.240456505
PAUPAR	3.19705	3.05306	0.802292	0.799596	-1.839814424
PDCD4-AS1	2.8896	4.04481	0.743526	0.444591	-2.361664954
PLAC1	5.3609	20.8042	4.65032	2.56626	-1.829803492
PRH1-PRR4	3.94288	3.8494	2.45845	2.63E-09	-1.588021793
PRKAG2-AS1	3.61614	4.40082	0.548942	1.31242	-1.995006862
RAPGEF3	2.33703	12.1243	0.227267	0.809831	-3.566984286
RARB	9.47423	11.8227	2.91186	3.50197	-1.700572773
RIMS3	4.99435	14.3845	2.62946	3.31978	-1.670816008
RNVU1-20	6.36995	7.07449	0	0	-6.092169374
RTCA-AS1	3.6821	3.46184	1.71497	0	-1.939232481
S100P	9.46726	82.0577	0	2.37745	-5.153298024

SCARNA1	58.1328	6.46082	15.7472	0	-2.022548624
SCARNA18	20.9778	0	0	0	-6.726408916
SCARNA21	34.1096	94.0724	0	0	-9.326227226
SCARNA3	0	28.479	0	0	-7.16385091
SCARNA8	0	27.2712	0	0	-7.101776122
SEPP1	13.6892	21.877	5.54941	3.85351	-1.897051783
SERPINF1	12.3666	22.6257	5.52954	4.20782	-1.824325269
SLC16A4	0.966833	5.86392	0.444314	0.209811	-3.041160085
SLC7A3	9.40735	31.3963	0.661833	3.16651	-3.347493905
SMIM14	2.58057	5.18163	0.690926	1.37706	-1.811755376
SNAR-D	54.3972	0	0	0	-8.092683155
SNORA14A	0	23.1066	0	0	-6.864594747
SNORA18	281.034	128.875	0	72.6515	-2.492976822
SNORA25	117.097	25.775	0	0	-9.482525641
SNORA2B	0	19.7751	0	0	-6.642058915
SNORA30	55.7515	0	0	0	-8.128032999
SNORA31	26.2704	86.6709	0	0	-9.143909938
SNORA33	44.305	24.3903	0	0	-8.428261756
SNORA38	1381.75	850.574	237.211	145.303	-2.544337103
SNORA4	125.525	0	0	0	-9.296055838
SNORA41	0	25.775	0	0	-7.020979939
SNORA45B	49.5769	0	0	0	-7.959332575
SNORA46	79.5511	0	0	0	-8.639360608
SNORA58	4.57408	1.72025	0	0	-5.02110879
SNORA5B	23.4195	25.775	0	0	-7.948206599
SNORA5C	17.9321	39.5502	0	0	-8.171984881
SNORA68	6579.29	4682.93	1349.61	1445.39	-2.01049344
SNORA71E	18.8745	0	0	0	-6.57550143
SNORA75	35.8642	0	0	0	-7.494423611
SNORA79	30.9471	0	0	0	-7.282954035
SNORD12B	225.026	0	0	0	-10.13715767
SNORD16	0	341.846	0	0	-10.73997464
SNORD22	839.112	368.617	0	206.148	-2.549384298
SNORD46	1961.4	2153.23	0	0	-14.32854521
SNORD60	3832.99	4264.22	0	0	-15.30517291
SNORD67	104.123	0	0	0	-9.026841547
SNORD97	3396.32	3098.17	1277.31	887.143	-1.58512489
SORCS1	2.28076	7.18794	0.605073	1.04786	-2.38351121
SUMO4	4.54554	2.87666	0.656908	0.785184	-2.214672506
TAS2R19	12.5817	9.41079	4.92803	1.96398	-1.645805072
TMEM255A	3.174	5.58039	1.66477	0.883281	-1.704186376
TNC	4.51033	14.9829	2.20211	1.85351	-2.210258517
TNFRSF9	0.987818	6.56029	0.177059	0.211774	-3.717913632
TXNIP	19.4676	34.7134	5.30821	3.39572	-2.610588525
TYMSOS	4.12365	4.23969	1.01227	1.21014	-1.821730541
UBE2Q2L	4.58083	4.15778	0	2.46273	-1.747144355

UNC5B	17.7357	22.6542	6.39352	5.99537	-1.688969704
VLDLR	6.20557	10.3472	1.92934	1.57881	-2.17562815
ZSCAN18	4.83397	15.6897	3.71332	2.90218	-1.60438811
